# Editorial: Perspectives in the use of hyaluronic acid in genitourinary diseases

**DOI:** 10.3389/fsurg.2026.1935349

**Published:** 2026-08-18

**Authors:** Marilena Gubbiotti

**Affiliations:** Department of Urology, Valdarno Hospital, USL Toscana Sud- Est, Montevarchi, Italy

**Keywords:** epithelial barrier, genitourinary disease, glycosaminoglycans, hyaluronic acid, regenerative medicine

## Introduction

The landscape of genitourinary medicine is rapidly evolving. Traditionally, the management of chronic urological and gynecological disorders has relied on disease-specific strategies aimed at controlling symptoms, reducing inflammation, or preventing recurrence. While these approaches have undoubtedly improved patient outcomes, they often address the consequences of tissue injury rather than the biological mechanisms driving disease onset and persistence. Increasing evidence indicates that several apparently distinct genitourinary conditions, including recurrent urinary tract infections (rUTIs), chronic prostatitis/primary prostate pain syndrome (CP/PPPS), radiation-induced cystitis, Bacillus Calmette–Guérin (BCG)-induced cystitis, and selected gynecological disorders, share common pathophysiological features, including epithelial barrier dysfunction, chronic inflammation, altered tissue permeability, and impaired mucosal repair (1–3).

Regenerative medicine is commonly associated with stem cell therapies, tissue engineering, and advanced biological technologies. However, regeneration also encompasses therapeutic strategies aimed at preserving tissue integrity and promoting endogenous repair mechanisms. Within this broader perspective, restoration of epithelial barrier function has emerged as a promising approach to interrupt the inflammatory cascade underlying many chronic genitourinary disorders (4).

Among the molecules investigated in this field, hyaluronic acid (HA) has gained increasing attention because of its biological role as a major component of the extracellular matrix and the glycosaminoglycan (GAG) layer. Beyond providing structural support, HA contributes to tissue hydration, modulation of inflammatory responses, epithelial repair, and maintenance of mucosal integrity, supporting restoration of epithelial integrity and functional recovery across different anatomical districts of the genitourinary tract (2–5).

This Research Topic was conceived to explore these evolving concepts through original investigations and expert perspectives spanning different clinical settings. Although the strength of evidence differs across the various clinical conditions discussed, the included studies collectively suggest that epithelial barrier dysfunction may contribute, to different extents, to disease pathophysiology and represent a potential therapeutic target in selected genitourinary disorders. Despite addressing diverse pathological conditions, the included studies converge on the hypothesis that disruption of epithelial homeostasis may reflect a shared biological framework linking chronic inflammation, recurrent infection, pain, and tissue dysfunction. Consequently, the findings included in this Research Topic support the concept that HA may be viewed not only as a disease-specific therapeutic option, but also as part of a broader mechanism-based strategy in which restoration of epithelial integrity may represent an important therapeutic target. This barrier-centered perspective provides the conceptual framework that connects the contributions included in this Research Topic and offers a contemporary interpretation of the expanding role of hyaluronic acid in genitourinary medicine.

## A Common Biological Target Across Different Clinical Settings

The studies included in this Research Topic collectively suggest that restoration of epithelial integrity may represent a shared therapeutic target across apparently distinct genitourinary disorders. Rather than focusing on individual diseases, they emphasize how preservation of the epithelial barrier may influence chronic inflammation, tissue repair, symptom burden, and ultimately patients' quality of life.

This concept is particularly evident in conditions characterized by urothelial injury. The original prospective multicentre study on rUTIs evaluated intravesical HA and chondroitin sulfate (CS) instillation protocols, showing a significant reduction in infection recurrence and improvement in lower urinary tract symptoms (LUTS), while suggesting that a simplified instillation schedule may achieve outcomes comparable to more intensive treatment protocols, consistent with previous evidence supporting restoration of the urothelial barrier (6). Similarly, the management of treatment-related bladder damage emerges as another important field for barrier-restoring therapies. The original study on symptomatic radiation cystitis showed that intravesical administration of adelmidrol and sodium hyaluronate improved pain, urgency, and hematuria, while the accompanying Opinion article on BCG-induced cystitis highlighted the importance of preserving urothelial integrity to improve treatment tolerability and maintain adherence to intravesical immunotherapy. This concept is also supported by previous clinical experience investigating HA and CS as adjunctive therapy for BCG-induced chemical cystitis (7).

A complementary perspective is provided by the original prospective study on CP/PPPS together with the accompanying Opinion article. Together, these two contributions combine clinical evidence with biological rationale, supporting the hypothesis that GAG replenishment may contribute to the modulation of chronic inflammation, epithelial dysfunction, and pain in a condition where effective therapeutic options remain limited, consistent with current EAU Guidelines on Chronic Pelvic Pain (8).

Finally, the prospective study on symptomatic cervical ectopy following vaginal hyaluronic acid therapy extends this concept beyond the urinary tract, suggesting that promotion of epithelial repair may represent a common therapeutic strategy across different genitourinary tissues. Collectively, these contributions support a mechanism-based interpretation of HA therapy, in which restoration of barrier function may represent a shared biological framework linking diverse clinical conditions.

## Future Directions: Moving from Clinical Evidence to Mechanism-Based Therapies

Although the evidence supporting HA in genitourinary diseases continues to expand, current studies remain limited by relatively small sample sizes, predominantly non-randomized study designs, heterogeneous treatment protocols, and, in several cases, the evaluation of combination products. Consequently, several important questions remain unanswered. In particular, the wide variability in formulation, dosage, route of administration, induction schedules, and maintenance strategies makes direct comparison between studies difficult and highlights the need for greater standardization in future clinical research.

Another key aspect concerns patient selection. It is increasingly evident that epithelial barrier dysfunction is unlikely to contribute equally to every patient or every stage of disease. Future investigations should therefore focus on identifying clinical and biological markers capable of recognizing those individuals who are most likely to benefit from barrier-restoring therapies. Such an approach would facilitate a transition from empirical treatment toward a more personalized and mechanism-based therapeutic strategy.

The growing interest in combination therapies also deserves attention. Several studies included in this Research Topic evaluated HA together with other bioactive compounds, such as CS, adelmidrol, curcumin, or quercetin, suggesting that modulation of multiple biological pathways may further enhance tissue repair and control of chronic inflammation. Whether these combinations provide additive or synergistic benefits remains to be established through comparative studies.

Ultimately, future research should focus on standardized treatment protocols, adequately designed comparator-controlled studies, clinically meaningful endpoints, and reliable biomarkers to better define the role of these therapies in personalized management of chronic genitourinary diseases.

Taken together, the contributions included in this Research Topic support a barrier-centered perspective that may guide future mechanism-based therapeutic strategies across selected genitourinary disorders.
